# Dosimetric evaluation of an atlas‐based synthetic CT generation approach for MR‐only radiotherapy of pelvis anatomy

**DOI:** 10.1002/acm2.12501

**Published:** 2018-11-26

**Authors:** Reza Farjam, Neelam Tyagi, Joseph O. Deasy, Margie A. Hunt

**Affiliations:** ^1^ Department of Radiation Oncology Weill Cornell Medicine/New York‐Presbyterian Hospital New York NY USA; ^2^ Department of Medical Physics Memorial Sloan Kettering Cancer Center New York NY USA

**Keywords:** bone‐ and fat‐suppressed CT, generalized registration error, pelvis anatomy, synthetic CT

## Abstract

**Purpose:**

To investigate the potential of an atlas‐based approach in generation of synthetic CT for pelvis anatomy.

**Methods:**

Twenty‐three matched pairs of computed tomography (CT) and magnetic resonance imaging (MRI) scans were selected from a pool of prostate cancer patients. All MR scans were preprocessed to reduce scanner‐ and patient‐induced intensity inhomogeneities and to standardize their intensity histograms. Ten (training dataset) of 23 pairs were then utilized to construct the coregistered CT‐MR atlas. The synthetic CT for a new patient is generated by appropriately weighting the deformed atlas of CT‐MR onto the new patient MRI. The training dataset was used as an atlas to generate the synthetic CT for the rest of the patients (test dataset). The mean absolute error (MAE) between the deformed planning CT and synthetic CT was computed over the entire CT image, bone, fat, and muscle tissues. The original treatment plans were also recomputed on the new synthetic CTs and dose–volume histogram metrics were compared. The results were compared with a commercially available synthetic CT Software (MRCAT) that is routinely used in our clinic.

**Results:**

MAE errors (±SD) between the deformed planning CT and our proposed synthetic CTs in the test dataset were 47 ± 5, 116 ± 12, 36 ± 6, and 47 ± 5 HU for the entire image, bone, fat, and muscle tissues respectively. The MAEs were 65 ± 5, 172 ± 9, 43 ± 7, and 42 ± 4 HU for the corresponding tissues in MRCAT CT. The dosimetric comparison showed consistent results for all plans using our synthetic CT, deformed planning CT and MRCAT CT.

**Conclusion:**

We investigated the potential of a multiatlas approach to generate synthetic CT images for the pelvis. Our results demonstrate excellent results in terms of HU value assignment compared to the original CT and dosimetric consistency.

## 1 INTRODUCTION

The use of magnetic resonance imaging (MRI) for radiotherapy application has been rapidly increasing in recent years.^1^ The main advantage of MRI is superior soft tissue contrast that improves the delineation of target volumes and organs at risk (OARs). Despite this clear superiority for tissue contouring, there are concerns regarding errors introduced by mis‐registration between the diagnostic MRI and radiotherapy planning CT or differences in bladder and rectum filling even if the MRIs are acquired in radiotherapy position. The idea of making MRI as the primary image set for radiotherapy planning and synthesizing a CT from the MRI information eliminates this concern and has enabled MR‐only radiotherapy approaches.

Various methods for generating synthetic CT images for pelvic anatomy have been introduced in the literature.^2^, ^3^, ^4^, ^5^, ^6^, ^7^, ^8^, ^9^, ^10^ Among all these promising approaches, MRCAT (**MR** for **C**alculating **AT**tenuation) available on 3T Philips Ingenia platform^11^ is one of the few commercial products^11^, ^12^ being used in our clinic for MR‐only radiation therapy.^13^ MRCAT CT is generated from a 3D mDixon fast field dual echo sequence by creation of three distinct images: water only, fat only, and in‐phase MRI. These image series are utilized in a classification algorithm to provide soft tissue and bony clusters. These two clusters are further divided into water, adipose, cortical, and spongy bones. Each class of tissue is then assigned a bulk electron density.

Although, MRCAT has been successfully applied in the clinic,^13^ the algorithm is currently limited to Philips MR scanners only. An ideal synthetic CT generation method would be independent of MR vendor and/or MR sequence. The MRCAT algorithm is also currently limited to generate bones till L4 which is not ideal if there is an intent to treat nodes higher than L4 for some prostate cases. Hence, alternative, more generally applicable methods for synthetic CT generation are still needed.

In this study, we aim to investigate the potential of a multi atlas‐based approach originally developed for head and neck anatomy^14^ to generate synthetic CT images for patients undergoing radiotherapy for prostate cancer. Several steps in the original algorithm were modified to expand its use to pelvic anatomy. We compare the image characteristics and dosimetric results to those of the deformed planning CT as well as MRCAT CT.

## 2 MATERIALS AND METHODS

### 2.A Image acquisition

After obtaining IRB approval, 23 sets of CT and MR images were retrospectively selected from a pool of prostate cancer patients (aged 54–87) for whom mDixon‐based MRCAT CT scans were also available. No prior assumption was made in terms of image quality to select this patient cohort. All patients received radiation therapy in our institution with a prescription dose ranging from 25 to 72 Gy using either five fraction stereotactic body radiosurgery or conventionally fractionated intensity modulated radiotherapy. For seven patients, the external beam radiotherapy (25 Gy in five fractions) was administered following brachytherapy. All CT and MRI scans were acquired in the treatment position. CT scans (either Philips Healthcare, Cleveland, OH (*n* = 21) or GE Medical System, Chicago, IL, USA) were acquired in the helical mode with a tube voltage of 120 kV, slice thickness of 2.5 mm, matrix size of 512 × 512, and in‐plane pixel size of 0.9766 × 0.9766 mm^2^. MR scans were acquired on a Philips 3T (Philips Healthcare, Cleveland, OH) Ingenia system using a vender‐provided phased‐arrayed anterior and posterior coils, and included a dual fast field echo mDixon (in‐phase, out‐phase, fat, and water) sequence with TE1/TE2/TR = 3.3/4.6/6.07 ms, flip angle = 10^o^, slice thickness of 2.5 mm and in‐plane pixel size of ~1 mm^2^. All MR scans were acquired with 30 cm length in superior‐inferior direction limited superiorly to L4. Ten of 23 patients were randomly selected to create the atlas and the remaining patients were reserved for the test dataset. MR scans contained noticeable artifact in the most inferior and superior slices. These slices were removed from our atlas resulting to lack of data near those regions.

### 2.B Image preprocessing

**Figure 1:**
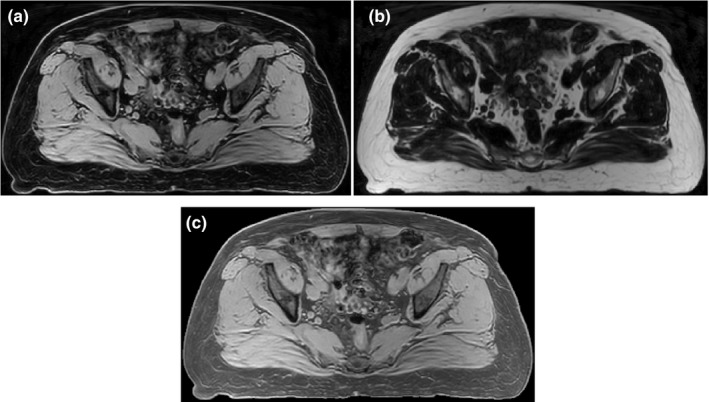
Example of water‐only (a), fat‐only (b), and fat‐enhanced water‐only image (c). As shown in (c), the contrast between fat and air regions was improved in fat‐enhanced water‐only image compared to water‐only image. Water‐only image was used to create the CT‐MR atlas and fat‐enhanced water‐only image was used for atlas propagation.

All MR scans were automatically preprocessed in two steps prior to synthetic CT generation. In the first step, an image analysis technique^15^ was utilized to reduce the intensity inhomogeneity due to field nonuniformity, tissue susceptibility effects, and scanner‐dependent variabilities. Local clustering properties of the image intensities were extracted using a model of intensity inhomogeneity surrounding each pixel to estimate the regional signal loss due to bias fields inhomogeneity. The original image was then corrected accordingly. This procedure was applied along the sagittal direction since this is the direction of more pronounced field inhomogeneity.^14^ In the next step, a landmark‐based standardization technique was used to standardize the MR intensity histogram. This reduced the scanner‐dependent variation in MR image intensities and facilitates the registration process. We applied the above procedure to water‐ and fat‐only images (Fig. 1). To find the tissue‐specific landmarks, fuzzy c‐means clustering was initially applied to the water‐ and fat‐only images to classify each image into dark and bright regions. Bright regions represent the muscle and fat tissues in the water‐ and fat‐only images respectively. The intensity histogram was then constructed for each cluster and the intensity corresponding to maximum histogram value was chosen as a landmark. This procedure produced two specific landmarks for each image. The local minimum between these two landmarks in the original histogram was used as the third landmark. After evaluation of the resulting histograms and consideration of the inherent contrast between muscle and bone in the water‐ and fat‐only images, a decision was made to use the water‐only images as the basis for the CT‐MR atlases and the subsequent atlas propagation. However, to further improve the contrast between the fat and air regions in the water‐only image, a linearly weighted component of the standardized fat‐only image was added to create a standardized fat‐enhanced water‐only image for use during the atlas propagation:

(1) $${\text{MR}}_{W,\mathrm{FE}}=\frac{{\text{MR}}_{S,W}+\alpha \cdot {\text{MR}}_{S,F}}{1+\alpha }$$

where in the above, MR_*S*,*W*_ and MR_*S*,*F*_ represent the standardized water‐ and fat‐only images and MR_*W*,FE_ denotes the standardized fat‐enhanced water‐only images [Fig. 1(c)]. *α *= 0.5 was used in this paper.

### 2.C CT‐MR atlas

**Figure 2:**
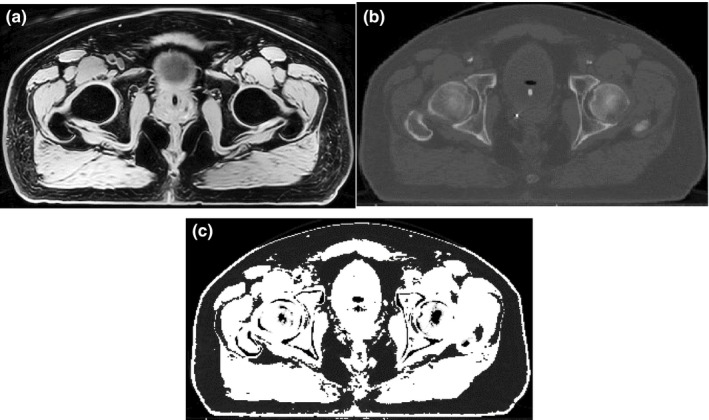
Example of a standardized water‐only image (a), planning CT (b), and fat‐ and bone‐suppressed CT (c). Bone and fat suppression was performed to improve the similarity in image contrast between the CT and water‐only MR images, thereby facilitating the deformation of the CT to the magnetic resonance imaging.

The CT number‐suppression approach developed in our previous synthetic CT method^14^ and used to improve similarity between CT and MR images was modified and applied to the CT images prior to deforming them onto the water‐only MR images for the atlas creation. Using thresholding (*H* < −250), air regions in CT were initially removed from the image. Using fuzzy‐c‐means (FCM) clustering, the remaining voxels were then automatically classified into fat, muscle, and bone. FCM is a supervised clustering algorithm assigning fuzzy membership to each data point corresponding to each cluster center based upon the distance between the cluster center and the data point. Bone regions were then suppressed by assigning air HU as described previously.^14^ The resultant CT images were then standardized such that the fat and muscle cluster centers on the CT histogram matched the intensity of the fat and muscle landmarks in the water‐only image. The resultant image is called CT_*S,*BS,FS_ where S, BS, and FS denote standardized, bone‐suppressed, and fat suppressed respectively (Fig. 2). Note that since the fat voxels in water‐only image have similar intensity to air, fat regions in the final CT are also suppressed after standardization [Fig. 2(c)].

To construct the CT‐MR atlas, ten of the 23 patients in the original dataset were randomly selected. For each patient, B‐spline deformable image registration (Plastimatch^16^) was used to deform the CT_*S,*BS,FS_ onto the standardized water‐only MR image. To expedite the registration, prior to deformation, the CT_*S,*BS,FS_ and water‐only MR images were initially aligned using the greater trochanters. During deformation, a subsampling rate of 1 × 1 × 1 was used to avoid smoothing and blurring effects. Mean square error was utilized as the cost function to fine‐tune the rigidly aligned images. The resulting deformation matrix was then applied to the original planning CT to obtain the deformed planning CT‐MR pairs (CT_reg_, MR_*W*,FE_) that form the atlas. It is worthwhile to note that the purpose of synthetic CT generation is to assign CT number to each MR voxel. Hence, MR geometry is our ground truth and we should deform everything onto the MR images.

### 2.D Synthetic CT generation for a new patient

For a new patient, the standardized fat‐enhanced water‐only MR image (MR_*W*,FE_) is constructed using the method described in Section 2B. All MR_*W*,FE_ images from the atlas pairs are then deformed onto the new patient's MR_*W*,FE_ using a rigid alignment followed by a B‐spline deformable registration similar to what is discussed in Section 2C. Using the resulting displacement matrices, the corresponding CT_reg_ from each CT_reg_‐MR_*W*,FE_ pair is subsequently transformed onto the new patient's MR_*W*,FE_. This procedure continues until all CT_reg_‐ MR_*W*,FE_ pairs are deformed onto the new patient's MR_*W*,FE_. After the images from all atlas pairs have been propagated onto the new patient, the local generalized registration error (GRE) metric is calculated between the fat‐enhanced water‐only MR images for the new patient and each of the deformed atlas pairs. GRE is a metric measuring the local registration error between a pair of co‐registered standardized MRIs by calculating the geometrical mean of the squared mean, variance, and entropy of the difference map of the two MR images over a small patch surrounding the voxel of interest. A 2‐D search with a radius of 2 mm, presented in^14^ is also performed around each voxel to find the best neighboring match for each atlas pair. Finally, the synthetic CT value at each voxel is a 1/GRE‐weighted average of the CT numbers from all atlases.

### 2.E Evaluation of results

Two methods were used to evaluate the performance of the proposed approach to generate synthetic CTs of the pelvic anatomy. In the first method, a dataset comprised of the ten patients whose CT‐MR pairs were used for atlas creation (training dataset) was used to generate synthetic CT for each patient in a leave‐one‐out scheme through which we also incorporated the training dataset into evaluation as well. Each patient was sequentially considered as a “new” patient, a CT‐MR atlas was formed from the remaining nine patients, and a synthetic CT was generated using the “new” patient water‐only MR image. In the second method, synthetic CTs were generated for the remaining 13 of the original 23 patients (test dataset) as an independent validation. For these patients, the CT‐MR atlas generated from the first ten patients was applied. As mentioned, for all 23 patients, the mDixon‐based MRCAT CTs were also available.

To quantify the voxel‐level accuracy of the intensities for each synthetic CT generated for evaluation, the mean absolute error (MAE) between the synthetic CT and its corresponding deformed planning CT (CT_reg_) and MRCAT CT scan, was computed over the entire image, as well as the bone, fat, and muscle regions separately. To identify the evaluation region for the entire image, a mask was applied to exclude the background from analysis. To obtain the bone, fat, and muscle regions, the corresponding clusters from the MRCAT CT images were identified and applied to the synthetic CT and CT_reg_ images. MRCAT has the same geometry as MRI and has an excellent classification result on fat and muscle. Cortical and spongy clusters were lumped together to produce the bony areas.

To evaluate the suitability of the synthetic CT for radiotherapy dose calculation, the patient's treatment plan, originally generated on the planning CT, was transferred to the deformed planning CT (CT_reg_), synthetic CT, and MRCAT CT and a forward dose calculation was performed (Eclipse V13.6, Varian Medical Systems, Palo Alto, CA). All patients’ original plans were either multifield intensity‐modulated radiotherapy or volume‐modulated arc therapy designed to treat the prostate bed, prostate with/without lymph nodes to doses ranging from 25 to 72 Gy with extreme hypofractionation (500 cGy × 5) or conventional fractionation. The dose–volume histograms (DVH) and dose statistics including the mean and maximum dose to the planning target volume (PTV) and OARs including the bladder, rectum, and urethra were compared for all plans. The quality of the digitally reconstructed radiographs (DRR) generated from the synthetic CT was also visually compared to those generated from the original planning CT and MRCAT CT. Also, to verify how good our proposed synthetic CT is for patient positioning, five patients with implanted fiducials were randomly selected from our patient list. Two‐dimensional kilovoltage image and cone beam CTs acquired during patient setup were then retrospectively and rigidly registered to the corresponding synthetic CTs. The displacement of the fiducial markers between both CTs were then measured to quantify the likelihood of the setup error using our proposed synthetic CT during patient positioning.

## 3 RESULTS

**Figure 3:**
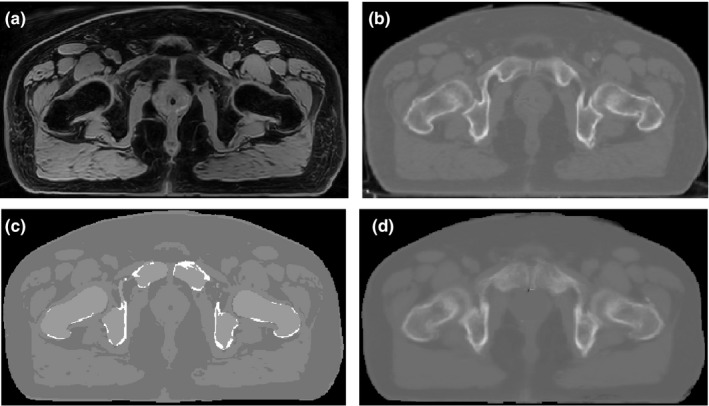
Example of a water‐only image (a) deformed planning CT (b) MRCAT CT (c), and synthetic CT (d) for a typical patient in the test dataset.

Figure 3 shows an example of a synthetic CT generated using the proposed approach along with the corresponding water‐only MRI, deformed planning CT (CT_reg_), and MRCAT CT for a typical patient from the test dataset. For this patient, the MAEs between the synthetic and deformed planning CTs were 54.67, 120.95, 43.43, and 52.52 HU for the entire CT, bone, fat, and muscle tissues respectively. The MAEs were 76.06, 185.17, 46.58, and 48.67 for the corresponding regions in the MRCAT CT scan. This indicates that the largest discrepancy between the synthetic and deformed planning CT exist in the bony structures.

**Figure 4:**
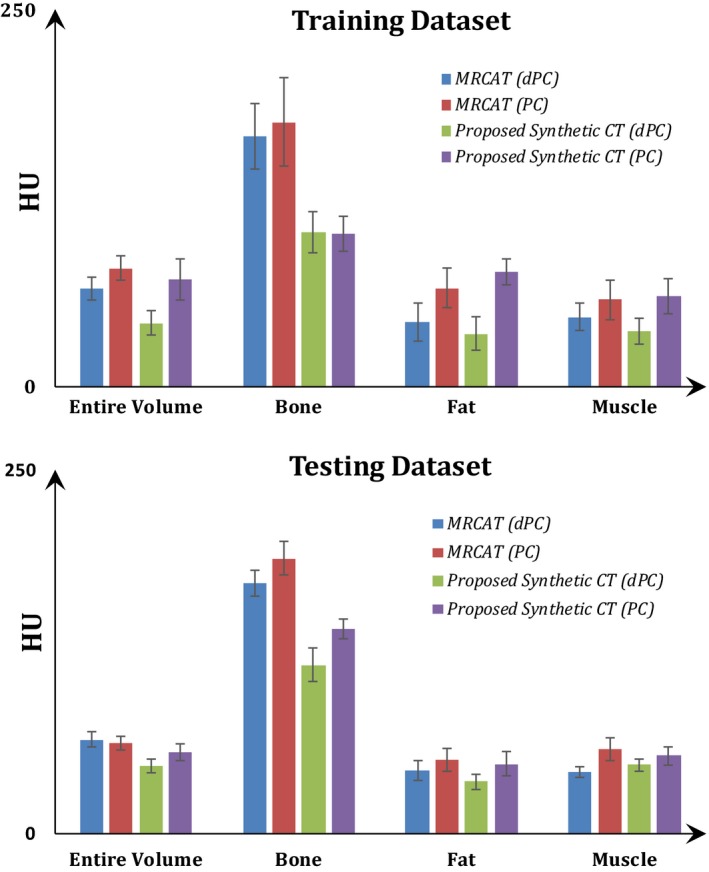
Mean absolute error (MAE) (mean ± SD) between the deformed planning CT/planning CT and the synthetic‐CT and MRCAT CT for different image regions. Results for the training and test datasets are given in the panels (a) and (b) respectively.

The average of mean absolute errors (MAE) between the deformed planning CTs and the synthetic CTs and MRCAT CTs across all patients are presented in Fig. 4 for both datasets. MAEs (mean ± SD) of 47 ± 5, 116 ± 12, 36 ± 6, and 47 ± 5 HU over the entire CT, bone, fat, and muscle tissue regions were observed for the synthetic CT in the test dataset. The MAEs were 65 ± 5, 172 ± 9, 43 ± 7, and 42 ± 4 HU for the corresponding regions in the MRCAT CT. Similar trends were observed in the training dataset [Fig. 4(a)]. For comparison, similar results obtained with the planning CT are also shown in Fig. 4. Bony structures were found to be the major areas of discrepancy for both MRCAT CT and the synthesized CT.

**Figure 5:**
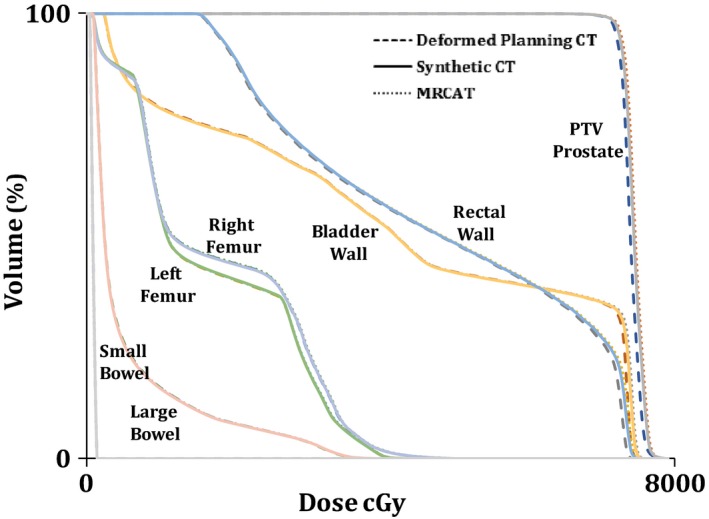
A dose–volume histograms (DVH) comparison for the planning target volume (PTV) and OARs for plans calculated on the deformed planning CT (Dash‐line), MRCAT CT (Dot‐line) and synthetic CT (Solid‐line) for a patient in the test dataset. Little difference is observed in the DVHs regardless of the image set used for calculation.

**Figure 6:**
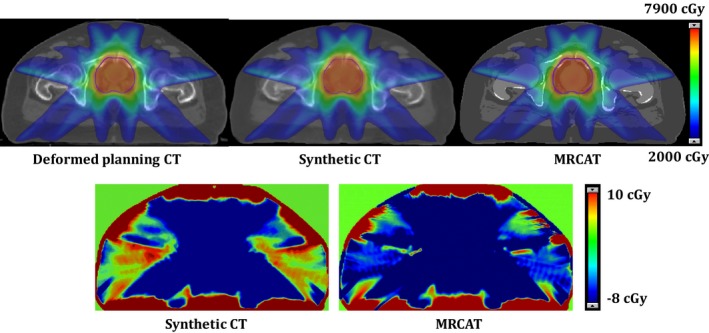
Example of dose distribution (Top row) for plans calculated using deformed planning CT, synthetic CT, and MRCAT CT along with dose difference maps between the deformed planning CT and the synthetic CT and MRCAT CT respectively.

**Figure 7:**
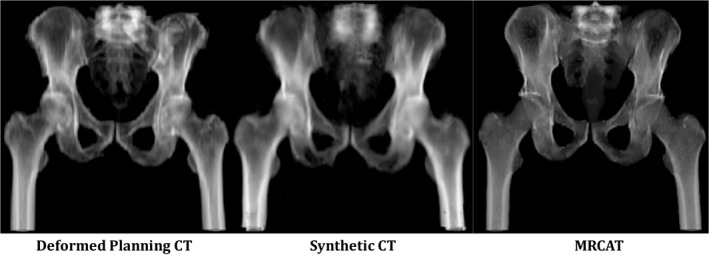
Example of digitally reconstructed radiograph (DRR) images corresponding to deformed planning CT (Left), our proposed Synthetic CT (Middle) and MRCAT CT (Right). The lower quality of the DRR in the superior region of the synthetic CT is mainly due to lack of data in our atlas in those regions. DVH summary and dose distribution for this patient are shown in Figs. 5 and 6 respectively.

Figure 5 illustrates a DVH comparison of the PTV and OARs for the deformed planning CT, synthetic CT, and MRCAT CT for a patient in the test dataset. Figure 6 shows dose distributions for the same patient overlaid on the axial slice at the isocenter for each plan. The dose difference maps between the deformed planning CT and the synthetic CT and MRCAT CT are also illustrated. As seen, a consistent dose distribution exists among all plans. The 2D gamma analysis of the isocenter dose distribution revealed the pass rate of 97.9%, 97.1%, and 92.7% for 3%/2 mm, 2%/2 mm and 1%/1 mm dose difference/distance to agreement criteria between the synthetic CT and deformed planning CT. These values were 97.9%, 97.1% and 90.6% for MRCAT CT and deformed planning CT, respectively. Also, Fig. 7 shows DRR images obtained using deformed planning CT, synthetic CT, and MRCAT CT, for this patient. Finally, our quantitative results showed that our proposed synthetic CT produce a robust positioning info with setup error of less than 1 mm in registering two CTs.

Tables 1 and 2 summarize the dose differences in the PTV and OARs between plans calculated using deformed planning CT, and synthetic CT or MRCAT CT for patients on the training and test datasets. For comparison purpose, the dose difference between the deformed planning CT and plan with no inhomogeneity correction were also included for the selected structures. As noted, the largest dose difference in the synthetic CT was less than 2.5% and seen in small bowel. Our further investigation revealed that this dose difference is mainly associated with regions receiving doses lower than 2 Gy which is clinically less significant. The 2D gamma analysis of the dose distribution in the axial plane through the isocenter revealed the pass rate of 96.86 ± 2.91% and 94.95 ± 0.81% for 1%/1 mm dose difference/distance to agreement criterion between the synthetic CT and deformed planning CT in the training and test dataset. These values were 94.9 ± 4.74% and 92.38 ± 3.67% between the MRCAT CT and deformed planning CT in the corresponding patient cohorts. For 2%/2 mm dose difference/distance to agreement criterion, the pass rate improved to 99.41 ± 0.56% and 98.94 ± 0.81% for the synthetic CT. The corresponding values were 98.51 ± 1.57% and 98.94 ± 1.31% for MRCAT CT. A pass rate of >99% was achieved for the synthetic CT and MRCAT CT for 3%/2 mm dose difference/distance to agreement criterion. Overall, these results show that the proposed synthetic CT approach can provide comparable or potentially even slightly better dose distributions compared to MRCAT CT. The results also reveal that there is no significant dose difference if we turn the heterogeneity correction off for dose calculation and we may end up having a discrepancy of ~4% over the target D_95_.

**Table 1:** The average of planning target volume (PVT) and various organs at risk (%) dose difference between the plan calculated using the deformed planning CT (CT_**reg**_) and the plans calculated using the synthetic CT or MRCAT CT for patients in the training dataset. NIC: No inhomogeneity correction

Training dataset		PTV prostate	Rectal wall	Bladder wall	Large Bowel	Small Bowel	Urethra
Synthetic CT	Max	0.7 ± 0.5	0.6 ± 0.4	1.1 ± 0.5	1.6 ± 1.2	2.3 ± 2.3	0.8 ± 0.5
	Mean	0.4 ± 0.5	0.8 ± 0.8	1 ± 0.8	2.1 ± 1.9	2.1 ± 2.6	0.5 ± 0.5
	D_95_	0.5 ± 0.5					
MRCAT CT	Max	0.8 ± 1	1 ± 0.9	0.9 ± 1	4.1 ± 5.8	2.7 ± 3	0.9 ± 0.7
	Mean	0.9 ± 1	0.8 ± 0.6	0.7 ± 0.6	2.4 ± 1.3	2.4 ± 2.7	0.7 ± 0.5
	D_95_	1.1 ± 0.9					
NIC	Max	0.98 ± 1.1	0.9 ± 1.1	0.8 ± 1	3.5 ± 5.2	3.3 ± 4	1.2 ± 1
	Mean	0.77 ± 1.1	0.6 ± 0.3	0.8 ± 0.7	2.8 ± 1.4	3.2 ± 3.6	0.9 ± 0.6
	D_95_	3.85 ± 1.8					

**Table 2:** The average of planning target volume (PTV) and various organs at risk (%) dose difference between the plan calculated using the deformed planning CT (CT_**reg**_) and the plans calculated using the synthetic‐CT or MRCAT CT for patients in the test dataset. NIC: No inhomogeneity correction

Test dataset		PTV Prostate	Rectal wall	Bladder wall	Large Bowel	Small Bowel	Urethra
Synthetic CT	Max	0.9 ± 0.3	0.9 ± 0.3	0.9 ± 0.2	1.9 ± 1.2	0.6 ± 0.2	0.7 0.1
	Mean	0.8 ± 0.2	0.8 ± 0.2	0.7 ± 0.5	0.8 ± 0.5	0.2 ± 0.2	0.9 0.4
	D_95_	0.9 ± 0.2					
MRCAT CT	Max	1 ± 0.5	1.6 ± 0.4	1.5 ± 0.3	1.5 ± 1.1	1 ± 0.7	1.5 ± 0.2
	Mean	1.3 ± 0.3	1.2 ± 0.3	1.2 ± 0.4	0.6 ± 0.4	0.8 ± 0.9	1.9 ± 0.2
	D_95_	1.4 ± 0.3					
NIC	Max	0.8 ± 0.6	1.4 ± 0.7	1.1 ± 0.5	1.3 ± 0.8	0.7 ± 0.3	1.3 ± 0.2
	Mean	1 ± 0.6	0.8 ± 0.5	0.9 ± 0.5	0.5 ± 0.4	0.5 ± 0.3	1.8 ± 0.2
	D_95_	3.3 ± 1.5					

## 4 DISCUSSION

In this work, we modified and extended a previously presented multiatlas approach^14^ to generate synthetic CT for pelvic anatomy. In this modified version, water‐only MRI, rather than in‐phase MRI, was used as the base image primarily to obtain superior image contrast between the bone and soft tissue in the pelvic region and to aid deformable image registration between CT and MR. To create the pelvis atlas, the first step was standardization of the water‐only MR image intensity histogram. Next, a bone‐ and fat‐suppression technique was applied to the original planning CT prior to deforming it to the standardized MR (Fig. 2). The purpose of the bone‐ and fat‐suppression was to generate a CT image for which the intensity of the fat, muscle and bony regions was similar to the corresponding regions in the standardized water‐only MR image. This greatly facilitated the deformation of the CT to the MR. Since in water‐only image, fat voxels have much lower intensity than they have in in‐phase image, fat‐suppression was also utilized to lower the intensity of the fat voxels in the CT as well. In addition, we used a landmark‐based rigid registration approach based on the location of greater trochanters to initially align the CT and MR, which also expedited the registration process. In fact, we use rigid registration as a starting point for B‐spline deformable image registration. Our empirical experiments showed that in the head and neck anatomy, the surface geometry of the head guides the rigid registration algorithm to provide an appropriate alignment. However, in the case of pelvic anatomy, the cylindrical surface geometry of the abdomen does not provide such guidance and the rigid registration may fail. Hence, we used landmark‐based alignment in this study, instead. It is also worthwhile to note that the result of CT to water‐only deformable image registration was not satisfactory without using landmark‐based alignment and bone and fat suppression technique in our study. To create the synthetic CT for a new patient, we followed similar steps explained previously.^14^ However, to facilitate MR‐to‐MR registration during the atlas propagation to a new patient, we blended a component of the fat‐only MR image to the water‐only MRI to create a fat‐enhanced water‐only MR image with superior fat‐to‐air discrimination. This was done for both the atlas and new patient water‐only MR images.

To evaluate the performance of the proposed methodology, the patient image sets were separated into a training dataset used for the atlas creation and a test dataset used for independent validation of the method. Synthetic CTs for the patients in the training set were generated using a leave‐one‐out approach. The synthetic CTs generated for both datasets were compared to MRCAT CT, a commercially available synthetic CT product, which is currently part of our routine clinical workflow for simulation of prostate patients. Overall, the dosimetric results using either synthetic CT or MRCAT CT (Figs 4, 5, 6, 7, Tables 1 and 2), were very similar and nearly identical to the original plan; indicating the suitability of either approach for clinical implementation. However, the synthetic CT method was slightly superior in terms of the Hounsfield Unit assignment. 2D gamma analysis indicated the potential for slightly better dosimetric results with synthetic CT which might be of value for certain patients or in regions of high dose gradient. Table 1 also shows that the largest dose discrepancies between the plan calculated on the synthetic CT and the original planning CT are for the small and large bowels and appears mostly in the regions receiving dose lower than 2 Gy but needs to be further investigated. If we assume that dose error of <1% in high dose region and <2% in general is sufficient, we may say that HU accuracy reported in this paper could be sufficient for treatment planning of the prostate cancer patient. Finally, it's worthwhile to say that in this study, we used only ten patients to construct our atlas. Our previous work has shown that this number of patients could be sufficient to produce acceptable synthetic CT. This could also be valid in pelvic anatomy as we have less variations and anatomical challenges such as air‐bone interface compared to head and neck anatomy. However, to determine the optimum number of patients in the training dataset is not a trivial task and should be thoroughly investigated. This will be accomplished at the time of clinical implementation of this approach.

One important feature of our proposed synthetic CT generation approach is that the algorithm is quite general, vender‐independent, and can be simply extended to different anatomical sites such as abdomen, lung, etc. Also, although it is originally developed based on mDixon sequence, the general procedure presented here is sequence‐independent and can be easily applied to other MR images. Furthermore, as mentioned, MRCAT is based on a model with a scan length of up to 30 cm till L4 which produces an issue in the case we need to treat higher nodes. However, we do not have such limitation in our proposed approach. We can include patients simulated with various position and scan length into our atlas and estimate the electron density for such cases.

Although the dosimetric results between the proposed synthetic CT method and MRCAT are promising, in some aspects, MRCAT performs more efficiently in clinical use. Foremost, the generation of the MRCAT CT at the scanner is complete within a few minutes of scanning the patient, while this is currently not the case for our approach. To implement our method, we used MEX function programming and Plastimatch in MATLAB R2017b on a four core Apple Mac Pro machine. With this setup, each deformation may take up to one hour. Each GRE and 2D search step may also take up to one hour for an image volume with a size of 512 × 512 × 120 pixels. Therefore, even with GPU programming, the entire process may require several hours to generate a single synthetic CT. Expediting the registration process and GRE calculation are important areas for future study. In addition, as shown in Fig. 3, MRCAT (and bulk density assignment approaches, in general) produce very sharp and clean images while the proposed atlas‐based approach generates a more blurred image. This may produce some difficulties if the synthetic CT is used as the primary image set for contouring of certain structures especially bony regions like femoral heads which are easier to contour on CT. Incorporating additional information from fat‐only and in‐phase images into generation of the synthetic CT may ultimately yield sharper images and is also an area for further investigation. Furthermore, the use of multiparametric GRE calculation is also part of our future study. Currently, we calculate the generalized registration error using the difference map between the two coregistered fat‐enhanced water‐only images. Applying the deformation matrices to all standardized MR images, including in‐phase, fat‐only, and water‐only image series, and utilizing them for similarity measurement, presumably provides more information to find the best match of a voxel in a new patient among the ones in the atlas.

## 5 CONCLUSIONS

We have modified and extended a previously described multiatlas approach focused on the head and neck region to generate synthetic CTs for pelvic anatomy. The results were compared to MRCAT CT, a commercially available product for radiotherapy use. The proposed multiatlas approach outperforms MRCAT in terms of Hounsfield Unit assignment and does slightly better in terms of reproducing the dose distribution from the original plan. This works demonstrates that our original atlas‐based method can be easily extended to other sites such as pelvis with promising dosimetric results. Computation time and blurriness of our final product are still challenges of our proposed method and require further investigation prior to clinical use.

## CONFLICT OF INTEREST

This work was partially supported through a Research Agreement from Philips Healthcare, Cleveland, OH.
